# Chromosomal radiosensitivity in breast cancer patients with a known or putative genetic predisposition

**DOI:** 10.1038/sj.bjc.6600628

**Published:** 2002-11-26

**Authors:** A Baeyens, H Thierens, K Claes, B Poppe, L Messiaen, L De Ridder, A Vral

**Affiliations:** Department of Anatomy, Embryology, Histology and Medical Physics, University of Gent, L. Pasteurlaan 2, B-9000 Gent, Belgium; Department of Medical Genetics, University of Gent, De Pintelaan 185, B-9000 Gent, Belgium

**Keywords:** breast cancer, genetic predisposition, chromosomal radiosensitivity, G2 assay, micronucleus (MN) assay, peripheral blood lymphocytes

## Abstract

The chromosomal radiosensitivity of breast cancer patients with a known or putative genetic predisposition was investigated and compared to a group of healthy women. The chromosomal radiosensitivity was assessed with the G2 and the G0-micronucleus assay. For the G2 assay lymphocytes were irradiated *in vitro* with a dose of 0.4 Gy ^60^Co γ-rays after 71 h incubation, and chromatid breaks were scored in 50 metaphases. For the micronucleus assay lymphocytes were exposed *in vitro* to 3.5 Gy ^60^Co γ-rays at a high dose rate or low dose rate. 70 h post-irradiation cultures were arrested and micronuclei were scored in 1000 binucleate cells. The results demonstrated that the group of breast cancer patients with a known or putative genetic predisposition was on the average more radiosensitive than a population of healthy women, and this with the G2 as well as with the high dose rate and low dose rate micronucleus assay. With the G2 assay 43% of the patients were found to be radiosensitive. A higher proportion of the patients were radiosensitive with the micronucleus assay (45% with high dose rate and 61% with low dose rate). No correlation was found between the G2 and the G0-micronucleus chromosomal radiosensitivity. Out of the different subgroups considered, the group of the young breast cancer patients without family history showed the highest percentage of radiosensitive cases in the G2 (50%) as well as in the micronucleus assay (75–78%).

*British Journal of Cancer* (2002) **87**, 1379–1385. doi:10.1038/sj.bjc.6600628 www.bjcancer.com

© 2002 Cancer Research UK

## INTRODUCTION

Breast cancer is the most common type of cancer in females, accounting for approximately 18% of all cancer cases in women worldwide (Parkin *et al*, 1988). One of the strongest and most consistently found risk factors for breast cancer is a family history of the disease (Teare *et al*, 1994). Out of all breast cancer patients 2% have a strong genetic predisposition, caused by highly penetrant genes (*BRCA1* and *BRCA2*) (Peto *et al*, 1999). As these highly penetrant predisposing genes cannot account for the overall increased risk in the relatives of breast cancer cases in general, it is suggested that a substantial proportion of breast cancer patients may be predisposed to breast cancer through mutations in low penetrance genes, which may be genes involved in the processing of DNA damage (Teare *et al*, 1994; Roberts *et al*, 1999; Scott *et al*, 1999; Burrill *et al*, 2000; Peto and Houlston, 2001).

Defects in DNA damage processing genes are likely to affect chromosomal radiosensitivity. In a large number of patients with inherited cancer-prone disorders such as ataxia-telangiectasia, Nijmegen breakage syndrome and hereditary retinoblastoma an enhanced chromosomal radiosensitivity has been demonstrated (Sanford *et al*, 1989; reviewed in Scott *et al*, 1999). More recently elevated chromosomal radiosensitivity has also been observed in significant proportions of patients with sporadic cancers with no obvious family history (Scott *et al*, 1994; Terzoudi *et al*, 2000). In breast cancer patients the elevated chromosomal radiosensitivity is confirmed in several independent studies (Scott *et al*, 1994, 1998; Parshad *et al*, 1996; Patel *et al*, 1997; Terzoudi *et al*, 2000; Baria *et al*, 2001a; Riches *et al*, 2001). In these studies the G2 assay, which involves the analysis of chromatid breaks in metaphase cells that are irradiated during the G2 phase of the cell cycle, was used to evaluate chromosomal radiosensitivity. Scott *et al* (1998, 1999) further demonstrated that breast cancer patients also show an elevated radiosensitivity with the G0-micronucleus (MN) assay. In the MN assay lymphocytes are irradiated in G0 phase, stimulated to divide, and micronuclei are scored in binucleate cells resulting from cytokinesis block. The fact that enhanced chromosomal radiosensitivity is also observed amongst blood relatives of breast cancer patients with high G2 and MN scores points to the heritability of chromosomal radiosensitivity in breast cancer (Knight *et al*, 1993; Roberts *et al*, 1999; Burrill *et al*, 2000). These findings support the view that enhanced chromosomal radiosensitivity of peripheral blood lymphocytes may be a marker for breast cancer predisposing genes of low penetrance.

The aim of our study was to investigate the chromosomal radiosensitivity by means of the G2 assay and the G0-MN assay in an extensive group (*n*=62) of breast cancer patients with a family history or early onset of the disease. A small number of these patients are carriers of a *BRCA 1/2* mutation. For the MN assay a standard dose is given at high dose rate (HDR) and at low dose rate (LDR). LDR was applied to allow repair and by this to discriminate in a better way between sensitive and non-sensitive individuals (Jones *et al*, 1995; Scott *et al*, 1998).

## MATERIALS AND METHODS

### Patients and normal controls

Heparinized blood samples were obtained by venepuncture from 60 normal healthy women, aged between 23 and 60 years (mean 37±12) and from 62 breast cancer patients, aged between 29 and 69 years (mean 45±10), during a period of 8 months. The study of the breast cancer patients was performed in collaboration with the Department of Medical Genetics, University Hospital Gent, Belgium. Patients are referred to genetic consultation because of familial or early onset breast cancer. Women fulfilling one of the following selection criteria were analysed for presence of a mutation in *BRCA1* and *BRCA2* as described (Claes *et al*, 1999a,b). (1) Three first degree relatives affected with breast and/or ovarian cancer (*n*=27). (2) Breast cancer patients from a family where at least in two first and/or second degree relatives breast and/or ovarian cancer is detected before the average age of 50 years (*n*=24). (3) Patients with bilateral breast cancer and both tumors diagnosed before an average age of 50 (*n*=8; five of them also fulfil criteria 1 while three of them fulfil criteria 2). (4) All patients diagnosed with breast cancer before the age of 35 years without a family history (*n*=11).

This group of breast cancer patients was selected for mutation analysis of the *BRCA1* and *BRCA2* genes, because a positive family history and/or diagnosis at young age is a significant risk factor for the development of hereditary breast cancer (Claes *et al*, 1999a,b).

The blood samples were collected at varying times after breast surgery and radio/chemotherapy (range 9 months to 21 years). However the majority of the samples were received 2–3 years after therapy. Data on the tumour stage, tumour type, chemotherapy, radiotherapy, oestrogen-receptor and progesterone-receptor status were also collected. In this study we performed the G2 and the MN assay on blood samples of the patients and on concurrent samples of healthy women.

All patients were given genetic counselling and signed an informed consent.

### The G2 assay

The G2 assay procedure of the Paterson Institute, Manchester (Scott *et al*, 1999) was followed with some minor changes. Briefly, heparinized blood was kept at ambient temperature before culturing, within 6 h after venepuncture. To a tissue culture flask (25 cm^2^) 0.5 ml of blood was added to 4.5 ml of complete culture medium consisting of RPMI-1640 medium supplemented with 10% foetal bovine serum (Life Technologies), 2% L-glutamine. Medium was warmed to 37°C and gassed (5% CO_2_/95% air) overnight in an incubator before adding to blood. A 10 μl of 1% PHA-P solution (Difco, Biotrading) was added as a mitogen. Per donor two cultures were set up: one for irradiation and one served as control. After 70–72 h incubation in a CO_2_ incubator at 37°C the cultures were irradiated with a dose of 0.4 Gy Co-60 γ rays at 37°C (Vral *et al*, 2002). At 30 min post-irradiation 75 μl colcemid (final concentration 0.15 μg ml^−1^; Sigma-Aldrich) was added, and 60 min later the cultures were arrested by putting them on ice for 5 min. For harvesting, blood cultures were transferred into centrifuge tubes and 5 ml of 0.075M KCl was added for 15 min, on ice. The cells were then fixed in cold (4°C) methanol : acetic acid (3 : 1). The fixed cells were kept in the refrigerator (4°C) for 48 h. For slide preparation, the cells were fixed once again with methanol : acetic acid (3 : 1), dropped onto clean dry slides and stained with 6% Romanowsky–Giemsa in HEPES buffer (pH 6.5) for 20 min. Fifty well spread metaphases were analysed for the appearance of chromatid breaks. The same slides were coded and analysed by two independent scorers. All types of single chromatid breaks were scored where a clear discontinuity was present (light microscopy, 1000 ×). Duplicate slides were made per sample and each scorer counted chromatid breaks in 25 metaphases on a different slide. No significant differences between the scorers were observed using a paired *t*-test (*P*>0.05).

### The G0-MN assay

Briefly, 0.5 ml of heparinized blood, always within 6 h after venepuncture, was diluted in 4.5 ml of complete culture medium in centrifuge tubes. The cultures were irradiated at 37°C with 3.5 Gy Co-60 γ rays at a high dose rate (HDR; 1 Gy min^−1^) or at a low dose rate (LDR; 4 mGy min^−1^) or sham-irradiated (Vral *et al*, 2002). Immediately after irradiation the lymphocytes were stimulated with 20 μl of 1% PHA-P solution (Difco, Biotrading) and 24 h later cytochalasin B (6 μg ml^−1^; Sigma-Aldrich) was added to block cytokinesis. Cells were harvested at 70 h after stimulation by a cold (4°C) hypotonic shock with 7 ml 0.075M KCl, followed by fixation in methanol : acetic acid : Ringer (0.9% NaCl) solution (10 : 1 : 11). The cells were stored overnight in the refrigerator (4°C) and fixed for another three times with methanol : acetic acid (10 : 1) (Vral *et al*, 1994). Suspensions of cells were dropped on clean slides and stained with 6% Romanowsky–Giemsa in HEPES buffer for 20 min. All slides were made in duplicate and coded. Per slide 500 binucleate cells (BN) were scored (light microscopy, 400×) according to the criteria of Fenech (1993). Each scorer analysed the number of MN in 500 binucleate cells on a different slide. In total 1000 binucleate cells were scored per sample. No significant differences between the scorers were observed using a paired *t*-test (*P*>0.05).

### Statistical analysis

For the comparison of the G2 and MN scores between different groups of breast cancer patients and controls, the unpaired Student *t*-test was applied. The differences in the yield of chromatid breaks or MN obtained for the same sample by two different scorers were analysed using a paired Student *t*-test. Differences in the proportions of sensitive patients and controls were compared using the chi-square test. The chi-square test was also used to compare the proportion of radiosensitive and non-radiosensitive patients treated with radio/chemotherapy, with positive oestrogen/progesterone receptor status and with tumour stage and grade. Correlations between parameters were assessed using Pearson correlation coefficient.

## RESULTS

### Reproducibility of the assays

Two of the healthy individuals were tested five times each with the G2 and the MN assay. For the G2 assay the average coefficient of variation (CV) obtained for these two controls (intra-individual variance) was 15% compared to a CV of 20% for inter-individual differences between control donors (*n*=51). For the MN assay the coefficient of variation for intra-individual differences was 9% at HDR and 10% at LDR, compared to a CV of 14% at HDR (*n*=53) and 17% at LDR (*n*=49) for inter-individual variance. A significant difference between the intra- and inter-individual variability was only obtained with the LDR MN assay (*P*=0.046; variance-ratio F test).

### Success rate of the assays

In this study the G2 assay and MN (HDR/LDR) assay were carried out on blood samples collected from 62 breast cancer patients and 60 healthy women. However, not all the G2 and MN cultures set up in this study were successful. Only those samples from which we could score 50 metaphases for the presence of chromatid breaks or 1000 BN cells for MN analysis, in both the irradiated and sham-irradiated cultures, were included in the study. The numbers of successful samples obtained with the G2 and MN assay are given in Tables 1

**Table 1:**
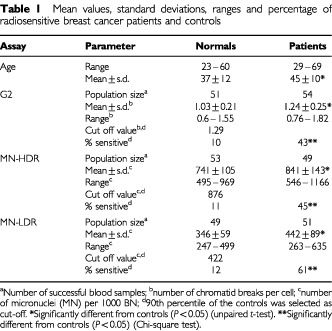
Mean values, standard deviations, ranges and percentage of radiosensitive breast cancer patients and controls

and 2

**Table 2:**
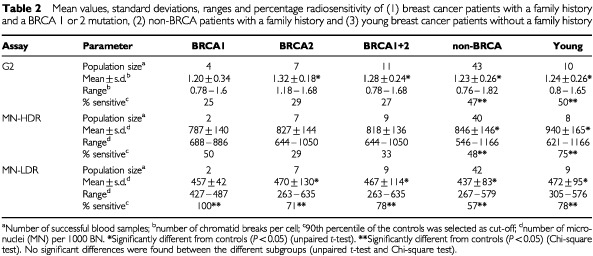
Mean values, standard deviations, ranges and percentage radiosensitivity of (1) breast cancer patients with a family history and a BRCA 1 or 2 mutation, (2) non-BRCA patients with a family history and (3) young breast cancer patients without a family history

and Figures 1

**Figure 1:**
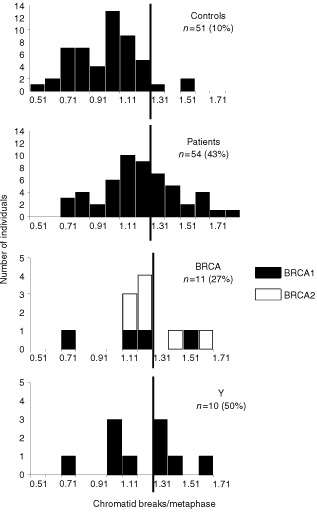
Radiation-induced G2 chromatid breaks in normal donors (Controls), all breast cancer patients (Patients), breast cancer patients with a BRCA1/2 mutation (BRCA), and breast cancer patients younger than 35 without a family history (Y). The vertical line represents the cut-off point between sensitive/non-sensitive.

,2

**Figure 2:**
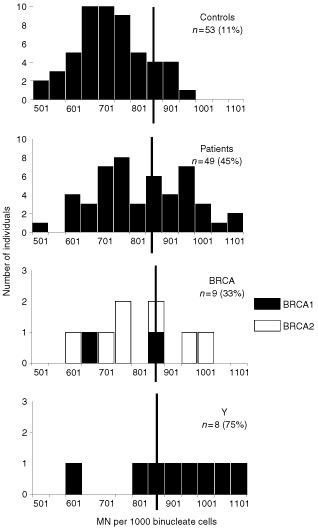
Radiation induced micronucleus yields after HDR irradiation for normal healthy donors (Controls), all breast cancer patients (Patients), breast cancer patients with a BRCA1/2 mutation (BRCA), and breast cancer patients younger than 35 without a family history (Y). The vertical line represents the cut-off point between sensitive/non-sensitive.

,3

**Figure 3:**
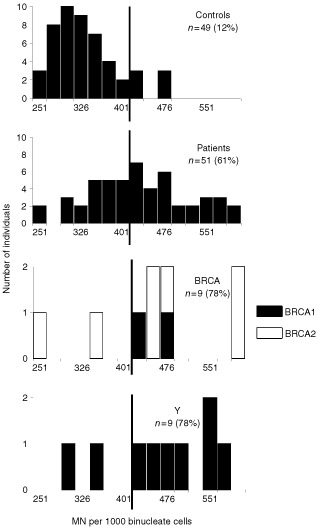
Radiation induced micronucleus yields after LDR irradiation for normal healthy donors (Controls), all breast cancer patients (Patients), breast cancer patients with a BRCA1/2 mutation (BRCA), and breast cancer patients younger than 35 without a family history (Y). The vertical line represents the cut-off point between sensitive/non-sensitive.

.

### The G2 assay

The results obtained with the G2 assay for the breast cancer patients and the control group of healthy women are summarized in Table 1 and presented graphically in Figure 1. The mean spontaneous yield was 0.05±0.05 (SD) chromatid breaks per cell and was not significantly different in both groups (unpaired *t*-test; *P*>0.05). For each sample the spontaneous yield was subtracted from the yield in irradiated cells to give the radiation induced yield. The mean yield of radiation induced chromatid breaks for the group of healthy women was 1.03±0.21 (SD) per metaphase. For the total group of breast cancer patients a mean radiation induced yield of 1.24±0.25 (SD) chromatid breaks per metaphase was obtained, which is significantly higher than for the control group (Table 1). Using the 90th percentile of the population of healthy donors as a cut-off value for radiosensitivity (1.29 chromatid breaks per metaphase) 43% of the breast cancer patients were radiosensitive compared to 10% of the healthy individuals. Comparing the G2 radiosensitive and non-radiosensitive patient group, there was no significant difference in age (unpaired *t*-test), tumour stage and grade, oestrogen/progesteron receptor positivity and previous radio- or chemotherapy (chi-square test) of the patients.

For further analysis we considered three subgroups in our total group of breast cancer patients: (1) a subgroup of patients that met criteria 1 and 2 (see Materials and Methods) and in which a mutation in *BRCA1/2* was detected (*BRCA1*, *n*=4; *BRCA2*, *n*=7); (2) a subgroup of patients that met criteria 1 and 2 but without a mutation in *BRCA1/2* (*n*=43); and (3) a group of young patients (age <35 year) without a family history and without a *BRCA1/2* mutation (*n*=10). The breast cancer patients with a *BRCA1/2* mutation were significantly more radiosensitive (μ=1.28±0.24 (SD)) than the controls but not significantly different from the other two subgroups (unpaired *t*-test, Table 2). Using the 90th percentile as a cut-off value, 27% of the breast cancer patients with a *BRCA1/2* mutation were radiosensitive. This was not significantly different from the control group (chi-square test, Table 2). For the group of young breast cancer patients without a family history and without a *BRCA1/2* mutation, the mean radiation-induced yield was 1.24±0.26 (SD) and five out of 10 young breast cancer patients had G2 values higher than the cut-off value (Table 2, Figure 1). This population was significantly more radiosensitive than the controls but not significantly different from the two other subgroups of breast cancer patients (unpaired *t*-test and chi-square test, Table 2).

### The G0-MN assay

The results obtained with the MN assay for the breast cancer patients and the age-matched healthy women are summarised in Table 1 and presented graphically in Figures 2 and 3. The mean spontaneous frequency of MN for all patients (26±17(SD) MN per 1000 BN) was significantly higher than that of the normals (15±10 (SD) MN per 1000 BN)(unpaired *t*-test; *P*<0.05). For each sample the spontaneous yield was subtracted from the yield in irradiated cells to give the radiation induced yield. The mean MN-induced yield for HDR and LDR irradiation for the controls was 741±105(SD) and 346±59(SD) MN per 1000 BN respectively. For the whole group of breast cancer patients we found a mean induced MN yield for HDR and LDR irradiation of 841±143(SD) and 442±89(SD) MN per 1000 BN respectively. These results are significantly higher compared to normal individuals for both the HDR and LDR MN assay (unpaired *t*-test, Table 1). Using the 90th percentile of the normals as the cut-off point for radiosensitivity, 45% of the patients were sensitive with the HDR MN assay (cut-off at 876 MN) and 61% of the patients were sensitive with the LDR MN assay (cut-off at 422 MN) (Table 1, Figures 2,3). With both MN assays the differences in proportion of radiosensitive patients and controls were significant (chi-square test, Table 1).

Selection of breast cancer patients with a mutation in *BRCA1* or *BRCA2* genes revealed a significantly higher mean value compared to the normal population only for the LDR MN assay. This mean value was, however, not significantly different from the mean values from the two other subgroups of breast cancer patients (unpaired *t*-test, Table 2). Taking the 90th percentile as cut-off, a high percentage (78%) had elevated LDR MN values, but this again was not significantly different from the other subgroups (chi-square test, Table 2).

For the subgroup of young breast cancer patients without a family history and without a *BRCA1/2* mutation, mean radiation induced MN yields of 940±165(SD) per 1000 BN for HDR and 472±95(SD) MN per 1000 BN for LDR were obtained. These mean values were significantly different from the controls but not significantly different from the other subgroups of breast cancer patients (unpaired *t*-test, Table 2). Although a high percentage of these patients are radiosensitive with the HDR (75%) and LDR (78%) MN assay (Table 2, Figures 2,3), this high proportion was not significantly different from the other patient groups (chi-square test, Table 2).

Concerning the clinical parameters, we found no significant differences between the HDR and LDR MN radiosensitive and non-sensitive patient group in age (unpaired *t*-test), tumour stage and grade, oestrogen/progesteron receptor positive status, and previous radio- or chemotherapy (chi-square test) of the patients.

### Correlations between the assays

**Figure 4:**
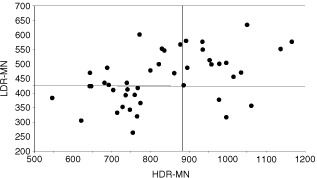
Correlation between induced MN yields after HDR and after LDR irradiation for breast cancer patients. The vertical line represents the cut-off point between sensitive/non-sensitive after HDR irradiation and the horizontal line represents the cut-off point between sensitive/non-sensitive after LDR irradiation.

To investigate the correlation between the different assays for radiosensitivity of the patient group the Pearson correlation coefficient was calculated. A poor correlation was found between G2 and G0 sensitivity using the same blood sample for both assays: G2 – HDR MN *r*=0.04 and G2 – LDR MN *r*=0.05. Only 14% of the patients were sensitive in the three assays (G2, HDR- and LDR MN). A good correlation was only obtained between the HDR MN and the LDR MN assay (*r*=0.46); 32% of the patients were sensitive in both assays (Figure 4

).

## DISCUSSION

We found that the studied population of breast cancer patients with a known or putative genetic predisposition is, for the mean value, more sensitive to ionising radiation than a population of healthy women both with the G2 as with the HDR and LDR MN assay. These results are in agreement with the enhanced chromosomal radiosensitivity observed in sporadic breast cancer patients (Scott *et al*, 1994, 1999; Terzoudi *et al*, 2000; Riches *et al*, 2001).

The lack of correlation between G2 and G0 chromosomal radiosensitivity observed in our study was also reported by Scott *et al* (1999) and points to the fact that different DNA damage processing mechanisms are operating in G0 and G2 phase of the cell cycle. At the molecular level two different repair pathways are described which are involved in the processing of DNA double strand breaks (dsb): homologous recombination (HR) and non-homologous end-joining (NHEJ) (Kanaar *et al*, 1998). NHEJ is more important for repairing γ-radiation induced dsb during G1-early S-phase, while HR is preferentially used for repair in late S-G2 phase (Takata *et al*, 1998; Rothkamm *et al*, 2001). Although recent studies have shown that G0/G1 chromosomal aberrations are essentially the result of misrepaired dsb (Wu *et al*, 1996; Boei *et al*, 2000; Fomina *et al*, 2000) by the NHEJ repair system (Jeggo, 1998; Takata *et al*, 1998; Rothkamm *et al*, 2001), the mechanisms involved in the formation of G2 chromatid breaks are not fully understood yet and different hypotheses have been proposed in literature (Parshad *et al*, 1996; Bryant, 1998; Terzoudi *et al*, 2000). These findings support the view that enhanced chromosomal radiosensitivity observed with the G2 and G0-MN assay in lymphocytes of a high proportion of breast cancer patients, cannot be due to a highly penetrant mutation in one gene, but may be due to low penetrance mutations in different genes involved in the processing of radiation induced DNA damage in G0 and G2 phase of the cell cycle.

Although the mean G2 and MN values are significantly higher in the studied breast cancer population compared to the normal population, there is an overlap between both groups. For this reason a cut-off value has to be determined, above which an individual can be considered as radiosensitive. This cut-off value is of course more or less arbitrary, but allows us to compare the proportion of radiosensitive individuals between different populations. As in the study of Scott *et al* (1999), the 90th percentile of the normal population was taken as cut-off point. For the G2 assay, the proportion of radiosensitive cases within our group of breast cancer patients with a known or putative genetic predisposition (43%), was comparable with the proportion of sensitive cases identified in a group of sporadic breast cancer patients (Scott *et al*, 1999). Our G2 data did not however confirm the findings of Parshad *et al* (1996) of a 2–3-fold increased yield of chromatid breaks in six out of seven familial breast cancer patients studied. For the MN assay a higher proportion of radiosensitive patients was observed with the LDR (61%) then with the HDR procedure (45%). Patients sensitive in both assays numbered 32%, and 82% of the patients that were sensitive at HDR were also sensitive at LDR (Figure 4). These results confirm the conclusion of Jones *et al* (1995) that the use of LDR irradiation allows better discrimination between controls and breast cancer patients than HDR irradiation. In the MN studies of Scott *et al* (1998, 1999) a much lower proportion of sporadic breast cancer patients was radiosensitive (27% with HDR-delayed stimulation; 15% with LDR). Apart from the population characteristics, differences in the MN assay protocols may also be responsible for the differences between the results of Scott *et al* (1998, 1999) and present work. The high percentage (61%) of radiosensitive cases found in our study with the LDR MN assay may point to the importance of defects in genes involved in the processing of radiation damage induced in G0 lymphocytes in breast cancer patients with a known or putative genetic predisposition.

Because in this study the blood samples of the patients were collected post radio/chemotherapy, we investigated the influence of previous therapies on the chromosomal radiosensitivity of the patients. No significant differences in chromosomal radiosensitivity were observed between the group of patients with and without therapy (*P*>0.05, unpaired *t*-test). These findings are in agreement with the data of Roberts *et al* (1999), who also found no significant differences between pre- and post-therapy G2 values in breast cancer patients. The mean spontaneous MN yield in the group of breast cancer patients was, however, significantly increased compared to the mean spontaneous MN yield of the controls. Taking into account an age dependent increase of 0.58 MN/year for a female population (Thierens *et al*, 2000), the observed increase in the mean spontaneous MN yield in the patient group cannot be attributed to the age effect alone, but may be partly due to the radio- or/and chemotherapy that some of the patients received before we collected the blood samples.

One subgroup considered in this study was the group of patients with a *BRCA1/2* mutation. Only with the LDR MN assay a higher proportion (78%) of this group of patients was found to be sensitive compared to the total group of breast cancer patients. Literature data concerning the chromosomal radiosensitivity in blood cultures of *BRCA1/2* patients are rare. Only in the study of Rothfuß *et al* (2000) the MN assay was performed on blood samples of *BRCA1* patients. According to these authors patients with a *BRCA1* mutation systematically have an enhanced micronucleus yield after an *in vitro* irradiation. Based on these results the authors even suggest the application of the MN-assay as a screening test for carriers of a *BRCA1* mutation in breast cancer families. The exact role of *BRCA1* and *BRCA2* in repair of DNA damage by ionising radiation is not yet fully understood (Abbott *et al*, 1998; Wang *et al*, 2001). *BRCA1* and *BRCA2* are linked with the RAD 52 epistasis group, which is involved in the homologous recombination repair pathway (Chen *et al*, 1999). Wang *et al* (2001) reported a lack of involvement of *BRCA1* and *BRCA2* in NHEJ. The fact that in our study only with the LDR MN assay a high proportion of *BRCA1/2* patients are radiosensitive cannot be explained by the involvement of *BRCA1/2* in homologous recombination. Although homologous recombination is preferentially used for repair in late S-G2 phase (Takata *et al*, 1998; Rothkamm *et al*, 2001) we could not demonstrate, with the G2 assay, that the group of patients carrying a *BRCA1/2* mutation is more radiosensitive compared to the total group of breast cancer patients. The proportion of radiosensitive patients within the *BRCA1/2* mutation group (27%) was lower than the proportion of sensitive cases identified in the total group of patients (45%), and not significantly different from the control group. These findings are partially in line with the findings of Baria *et al* (2001b). Using the G2 assay, they found no difference in G2 chromosomal radiosensitivity between healthy *BRCA1* mutation carriers and control individuals and they concluded that G2 radiosensitivity is no feature of individuals heterozygous for *BRCA1* mutations.

Analysis of the sub-population of young breast cancer patients without family history and without *BRCA1/2* mutation revealed that this subgroup is the most radiosensitive for all the different assays (G2 assay: 50%; HDR MN assay: 75%; LDR MN assay: 78%) (Table 2, Figures 2,3). No literature data are available at the moment concerning chromosomal radiosensitivity in young cancer patients. The elevated chromosomal radiosensitivity of this group of patients may suggest that these patients have a specific, still unknown, defect in DNA damage processing. Gentile *et al* (1999) suggest that these patients have unidentified genes involved in initiation and/or progression of breast cancer, situated on chromosome 11.

In conclusion our data show that breast cancer patients with a known or putative genetic predisposition are more radiosensitive than normal individuals both with the G2 and with the MN assay. The radiosensitivity is most pronounced using the MN assay after an irradiation at low dose rate. Out of the different subgroups considered, the young breast cancer patients without family history show the highest proportion of radiosensitive cases, and this in all the assays. Future studies will be focused on the group of breast cancer patients with a *BRCA1/2* mutation and young breast cancer patients without a family history.
